# Keeping It All Going—Complement Meets Metabolism

**DOI:** 10.3389/fimmu.2017.00001

**Published:** 2017-01-18

**Authors:** Martin Kolev, Claudia Kemper

**Affiliations:** ^1^Division of Transplant Immunology and Mucosal Biology, MRC Centre for Transplantation, King’s College London, Guy’s Hospital, London, UK; ^2^Laboratory of Molecular Immunology, The Immunology Center, National Heart, Lung, and Blood Institute (NHLBI), National Institutes of Health (NIH), Bethesda, MD, USA

**Keywords:** complement, metabolism, evolution, T cells, metabolic disease

## Abstract

The complement system is an evolutionary old and crucial component of innate immunity, which is key to the detection and removal of invading pathogens. It was initially discovered as a liver-derived sentinel system circulating in serum, the lymph, and interstitial fluids that mediate the opsonization and lytic killing of bacteria, fungi, and viruses and the initiation of the general inflammatory responses. Although work performed specifically in the last five decades identified complement also as a critical instructor of adaptive immunity—indicating that complement’s function is likely broader than initially anticipated—the dominant opinion among researchers and clinicians was that the key complement functions were in principle defined. However, there is now a growing realization that complement activity goes well beyond “classic” immune functions and that this system is also required for normal (neuronal) development and activity and general cell and tissue integrity and homeostasis. Furthermore, the recent discovery that complement activation is not confined to the extracellular space but occurs within cells led to the surprising understanding that complement is involved in the regulation of basic processes of the cell, particularly those of metabolic nature—mostly *via* novel crosstalks between complement and intracellular sensor, and effector, pathways that had been overlooked because of their spatial separation. These paradigm shifts in the field led to a renaissance in complement research and provide new platforms to now better understand the molecular pathways underlying the wide-reaching effects of complement functions in immunity and beyond. In this review, we will cover the current knowledge about complement’s emerging relationship with the cellular metabolism machinery with a focus on the functional differences between serum-circulating versus intracellularly active complement during normal cell survival and induction of effector functions. We will also discuss how taking a closer look into the evolution of key complement components not only made the functional connection between complement and metabolism rather “predictable” but how it may also give clues for the discovery of additional roles for complement in basic cellular processes.

## Introduction

Detection and subsequent removal or containment of microbes and pathogens, harmful entities and altered, dangerous self-derived antigens by the host’s immune system is key to survival. Particularly, the pattern-recognition receptors (PRR) of the innate immune system, including for example the toll-like receptors (TLRs), the inflammasomes, and the complement system are prime sensors of molecular patterns derived from pathogens or otherwise dangerous antigens (pathogen- or danger-associated molecular patterns, PAMPs or DAMPs) (1–3). Activation of these PPR systems not only induces an immediate strong protective effector response by innate immune cells such as neutrophils, monocytes, and natural killer T cells but also leads to the activation and instruction of adaptive immunity (2, 4, 5). Although it has long been appreciated that engagement of PPRs on and/or in cells and subsequent induction of their respective downstream effector pathways is required for the expression and secretion of cytokines, growth factors, cytotoxic molecules, and general activation of immune cells, what has been unexpected is that many of these events are driven by direct impact of PPRs on cell metabolism (6). Specifically, the emerging critical role for the complement system in the regulation of key metabolic pathways of cells has been surprising, as complement is commonly considered a serum-effective system with its functions mostly fully explored.

## Systemic, Autocrine, and Intracellular Complement in Protective Immune Responses

### Systemic- and Serum-Operative Complement

**Figure 1:**
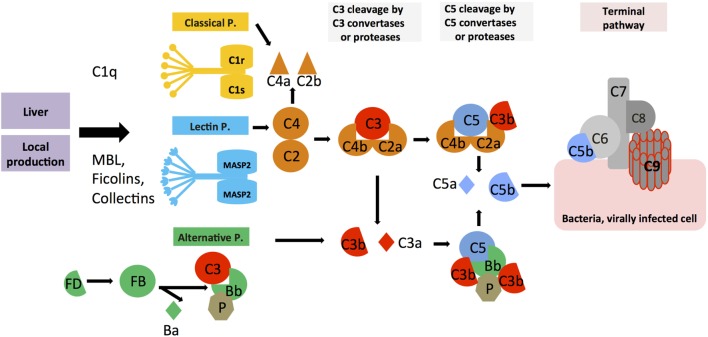
**Systemic complement activation**. Serum-circulating complement can be activated *via* three pathways: the classical, lectin, and alternative pathways, which all cumulate in the formation of multiprotein complexes termed C3 convertases. The classical and lectin pathway C3 convertases (C4bC2a) and the alternative pathway C3 convertase (C3bBb) lead to cleavage of C3 into the opsonin C3b and the anaphylatoxin C3a. Properdin (P) is a stabilizator of the alternative C3 convertase. Upon subsequent generation of C5 convertase (C4bC2aC3b for the classical and lectin pathways, C3bBbC3b for the alternative pathway), C5b and the anaphylatoxin C5a are produced, with surface-bound C5b initiating the formation and insertion of the terminal complement pathway (TTC; or membrane attack complex, MAC) on pathogens (or other target membranes).

The complement system was discovered by Jules Bordet and Paul Ehrlich at the end of the nineteenth century as a “system of serum-circulating proteins *complementing* antibody-mediated immune responses” (7, 8). The complement system that is composed of over 50 blood- and lymph-circulating, as well as membrane-bound, proteins is a central part of innate immunity and constitutes the first line of defense in the detection and removal of pathogens that have breached the host’s protective barriers. The complement proteins circulating in blood are majorly secreted by the liver and comprise the PRR components and the effector molecules, which exist mostly in inactive pro-forms. The system becomes activated in a cascade-like fashion when triggered through one or more of the three main activation pathways, the classical, the lectin, or the alternative pathway (Figure 1) (9, 10). The generated activation fragments then engage membrane-bound complement receptors and regulators expressed by cells, which in turn transmit instructive signals into the cell. Each activation pathway cumulates into the generation of the C3 and C5 convertase complexes, which cleave C3 into the bioactive opsonin C3b and the anaphylatoxin C3a, and C5 into C5b and the anaphylatoxin C5a, respectively. Deposition of C5b onto a pathogen surface seeds the generation of the pore-forming terminal complement complex (TCC; or membrane attack complex, MAC), leading to direct lysis of the microbe. Further, the generation of C3b leads to opsonization and phagocytic uptake of the targeted pathogen by scavenger cells (*via* engagement of receptors specific for C3 activation fragments), while C3a and C5a induce migration and activation of innate immune cells and drive a broad inflammatory reaction *via* engagement of their specific G protein-coupled receptors (GPCRs) (9, 11, 12). The critical role of serum-circulating complement as sentinel for pathogen invasion is underpinned by the fact that deficiencies in key complement components lead to severe and recurrent infections (13–15). Importantly, and similar to TLRs and the inflammasomes, serum-circulating complement-derived PRRs recognize not only PAMPs but also DAMPs. For example, ficolins and the C1 complex (C1q, C1r, and C1s) detect danger molecules produced by stressed and dying cells, such as surface blebs on apoptotic cells, and deficiencies or dysfunctions in C1 proteins are associated with the autoimmune disease systemic lupus erythematosus (SLE) (16, 17). Aside from its critical role in innate immune responses, complement also impacts heavily on adaptive immunity. Receptors specific for the fragments produced by complement activation transmit signals into various cells, including B and T cells (18, 19). During B cell receptor (BCR) activation, stimulation of complement receptor 2 (CR2, CD21) through C3d-coated antigen reduces the threshold for BCR signaling, thereby providing important costimulation for optimal antibody production (20, 21), and explaining why serum C3 deficiency often causes common variable immunodeficiency (22). Further, complement receptor signaling on B cells and follicular dendritic cells contributes to induction of B cell memory and maintenance of B cell tolerance (23, 24), and complement receptor activation on T cells is required for the induction of a range of effector functions (2, 11, 25, 26).

### Autocrine- and Intracellular-Operative Complement

**Figure 2:**
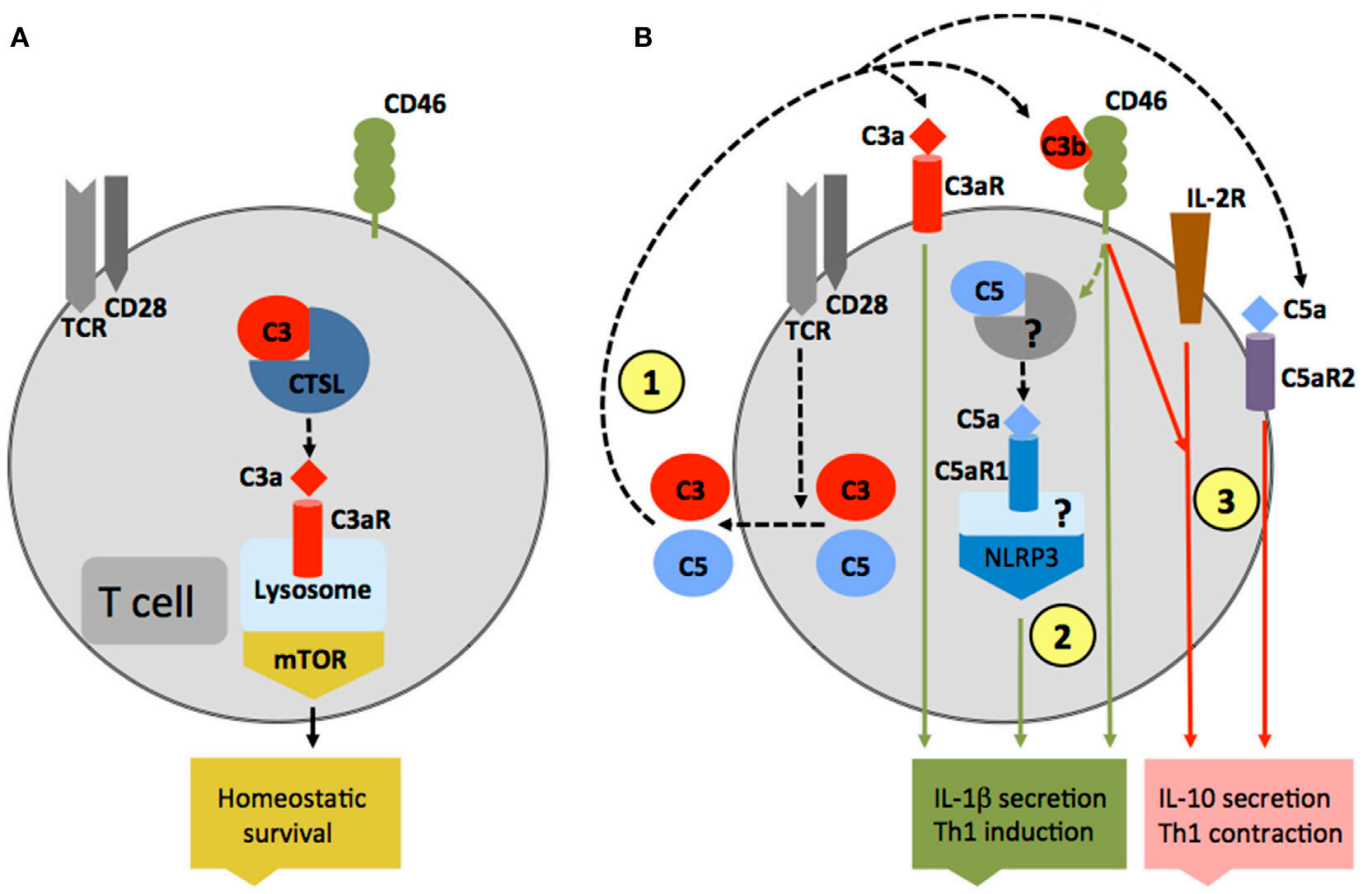
**Autocrine and intracellular complement activation**. **(A)** In resting T cells, C3 is processed intracellularly by the protease cathepsin L to generate bioactive C3a that sustains low-level mechanistic target of rapamycin (mTOR) activity *via* the engagement of the intracellular C3aR expressed on lysosomes and thus contributes to homeostatic survival of CD4^+^ T cells. **(B)** Local complement activation is triggered when activating signals [here, T cell receptor stimulation or toll-like receptors activation on antigen-presenting cells (not shown)] initiate secretion of preformed C3, C5, factor B, and factor D located in cellular storages, leading to C3 and C5 convertase formation in the extracellular space as well as on the cell surface—and the generation of C3a, C3b, C5b, and C5a (1). These complement fragments bind to their respective receptors on the T cell surface and induce Th1 induction with interferon (IFN)-γ secretion. C5 is also processed intracellularly by a yet unknown protease/convertase into C5a and C5b, and this process is increased through CD46-mediated signals. Intracellular C5a engages the intracellular C5aR1, which triggers NLRP3 inflammasome assembly and intrinsic IL-1β secretion that sustains Th1 induction in an autocrine fashion (2). Importantly, autocrine CD46 activation in conjunction with IL-2R signaling also induces IL-10 co-production in Th1 cells and the transition into a (self)regulative Th1 contraction phase (3). This “IL-10 switch” is accompanied by autocrine surface engagement of the C5aR2 *via* surface-shuttled C5a/C5a-desArg (3) through an as yet undefined signaling pathway (but possibly through direct suppression of intracellular C5aR1 activity, not shown).

Traditionally, the observed effects of complement receptor activation on various cell types was thought to be mediated solely by complement activation fragments generated in serum, the lymph, or interstitial fluids. However, particular work on the effects of complement on T cell responses demonstrated that complement activation fragments generated by antigen-presenting cells (APCs) and T cells themselves function in an autocrine and also unexpectedly in an intracellular fashion and are required for normal T cell immunity (27–31) (Figures 2A,B). For example, in resting human CD4^+^ T cells, activation of C3 occurs continuously within the cell through cleavage *via* the protease cathepsin L (CTSL) into bioactive fragments C3a and C3b. C3a generated by this “pathway” activates the C3aR located on lysosomes, and this process is required for homeostatic survival of T cells (Figure 2A) (31). T cell receptor (TCR) activation induces the rapid translocation of intracellular C3a and C3b to the cell surface where they engage surface C3aR and the costimulatory complement receptor/regulator CD46 (membrane cofactor protein, MCP), respectively, two events vitally required for interferon (IFN)-γ production and human T helper cell type 1 (Th1) induction (of note, serum-derived C3 cannot drive this response) (Figure 2B). Indeed, individuals lacking CD46 expression or C3 secretion by T cells have diminished Th1 immunity (but normal Th2 responses) and suffer from recurrent infections (32, 33). Conversely, uncontrolled intracellular C3 activation leads to dysregulation of CD46-mediated stimulation of human T cells and contributes to pathologically hyperactive Th1 responses in rheumatoid arthritis (RA) (33). Human CD4^+^ T cells also contain an intracellular C5 activation system, and the engagement of the intracellular C5a receptor C5aR1 (CD88) upon TCR stimulation is required for normal production of reactive oxygen species (ROS) and NLRP3 inflammasome-driven intrinsic IL-1β secretion that supports optimal and sustained Th1 induction during T cell migration into tissues (Figure 2B) (34). In mice [which do not express CD46 on somatic tissue (35)], activation of the anaphylatoxin receptors C3aR and C5aR1 expressed by T cells is also critical for induction of normal Th1 and Th17 activity, and also controls natural regulatory T (Treg) cell responses (30, 36, 37). T cells from mice deficient in the *C3ar* and *C5ar1* additionally show reduced survival in the periphery, further substantiating a role for these receptors in T cell homeostasis (30). Of note though, in the studies conducted on mouse T cells so far, a functional role for intracellular complement activation had not yet been addressed.

Autocrine-active complement is also needed for normal Th1 cell contraction (Figure 2B): after successful CD46-driven Th1 expansion, CD46 engages in an as yet undefined crosstalk with the IL-2 receptor and induces IL-10 co-production in Th1 cells that “switches” these cells into a (self)regulative contraction phase (33). Deviations in this crosstalk prevent normal IL-10 co-induction and contraction in Th1 cells and contribute to chronic disease settings, including multiple sclerosis (MS) (38) and RA (33). Further, autocrine surface engagement of the alternative C5aR2 (also known as C5L2 or GPR77) *via* intracellularly generated and then surface-shuttled C5a/C5a-desArg negatively regulates intracellular C5aR1 stimulation and, thus, also aids in Th1 contraction (Figure 2B). Although the biologic role of intracellular complement activation is currently best studied in human CD4^+^ T cells, it has been observed in all cells analyzed for far and is therefore likely of broad physiological relevance (31).

Thus, complement activation takes place at different locations and it is the location that dictates function: while serum-circulating complement is critical for its sentinel function in the recognition and removal of pathogens breaching host barriers, autocrine-functioning complement directs adaptive T cell and APC responses and tonically active intracellular complement may sustain cell homeostasis. Particularly, the latter concept aligns well with the growing appreciation that complement plays an unanticipated active role in tissue development, homeostasis, and repair. Although the function of intracellular complement and its connection with cell metabolism has not yet been investigated during tissue maintenance, complement-mediated signals are crucial for liver and retina regeneration after injury and for bone healing (39–42). For recent reviews into these unexpected “anti-inflammatory” complement activities, please see Ref. (2, 11, 43).

## Complement and Metabolism

The discovery of an intracellular complement system, the complosome, further substantiates the growing notion in the field that complement may not serve strictly classic immune-related functions but that this system may also control basic processes of the cell and particularly those of metabolic nature. Such a general and driving role for complement in cell metabolism could also explain its impressive and seemingly ever expanding range of effector functions.

### Systemic and Extracellular Complement in Metabolism

**Figure 3:**
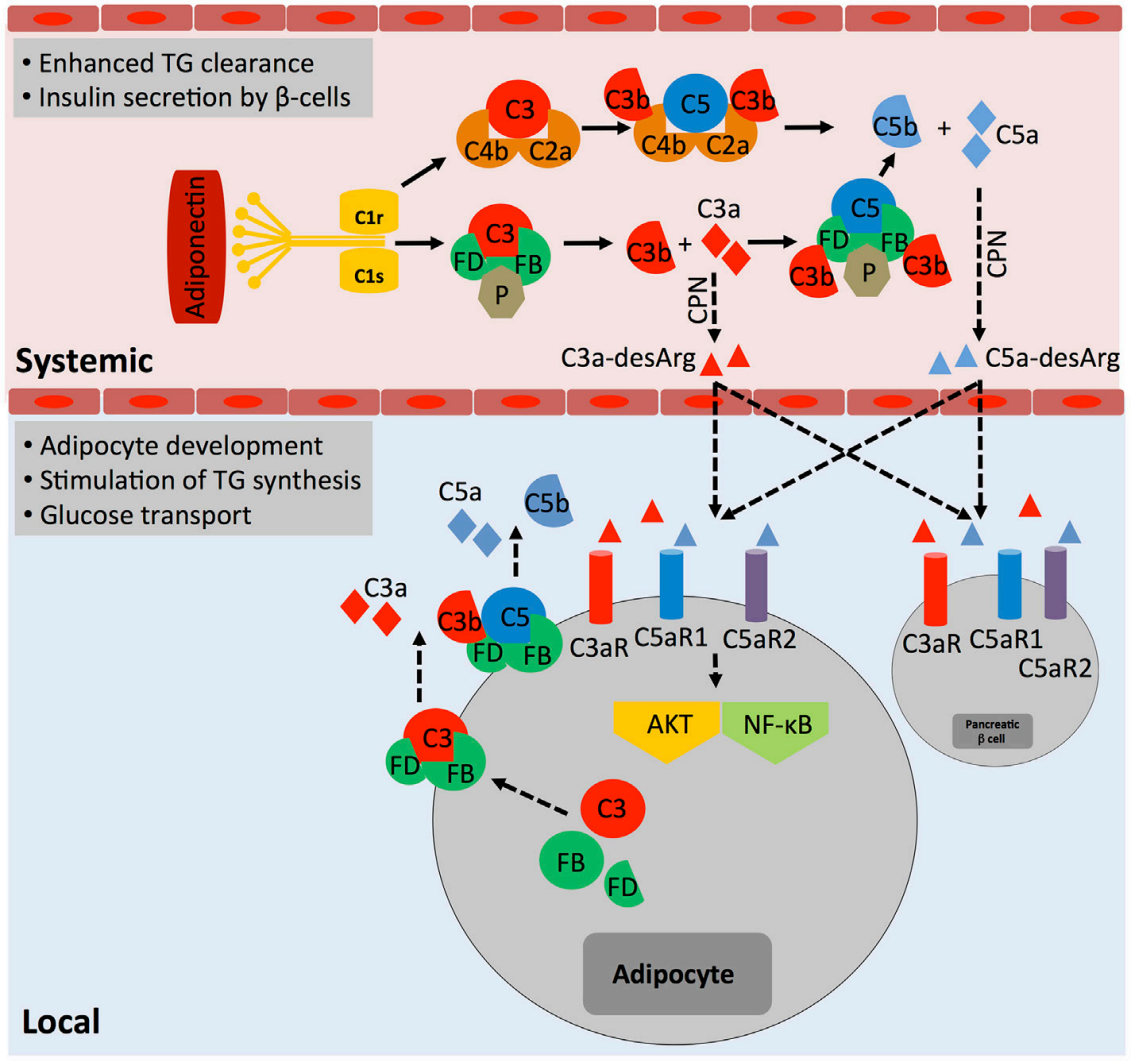
**The role of systemic complement in metabolism**. Metabolically active proteins can activate systemic complement in the absence of infection. For example, adiponectin activates directly C1q, which results in the generation of C3a and C5a fragments *via* classical pathway activation. The anaphylatoxins are further processed by carboxypeptidase N (CPN) to C3a-desArg and C5a-desArg, which then impact on triglyceride (TG) clearance, increase insulin resistance, and alter adiposity by affecting signaling through the three anaphylatoxin receptors C3aR, C5aR1, and C5aR2 in several cell types such as adipocytes and pancreatic β-cells *via* diffusion into respective tissues. Importantly, although not yet formally proven, locally generated and activated complement also likely affects adipocytes activity as these cells are a major source of Factor D and can also synthesize and secrete other complement proteins such as Factor B, C3, and C5. These proteins, when secreted, form extracellular C3 and C5 convertases and generate locally C3a and C5a fragments, which engage their respective receptors and trigger AKT and NF-κB signaling that contributes to adipocyte development and activity.

The idea that complement may aid in basic cellular processes is not new. In fact, a functional connection between complement activity in serum and regulation of lipid metabolism has already been made very early on, in the late 1980s when the desarginated form of C3a, C3a-desArg [initially identified as acylation stimulation protein (ASP)], was shown to stimulate triglyceride (TG) accumulation and glucose transport in adipocytes through interaction of C3a-desArg with the C5aR2 (44). Although C5a and C5a-desArg, verified binding partners to C5aR2, seem to play a subordinate role in these processes (45), recent data suggest that C3a-desArg is unable to bind C5aR2 (46) and hence the mechanism by which C3a-desArg mediates TG regulation operates highly likely rather through C3aR signaling. Generation of metabolism-modulating C3a-desArg is mostly dependent on alternative pathway activation because both C3-deficient and factor B (FB)-deficient mice exhibit decreased glucose tolerance and delayed clearance of TG and non-esterified fatty acids when compared with wild-type mice (47). Plasma levels of C3a-desArg are also increased in obese individuals and in patients suffering from diabetes type II (11, 48). However, increased or uncontrolled activation of the classical complement pathway also contributes to dysregulated TG clearance as C1q binding to adiponectin was observed in obesity and could further augment C3a-desArg levels *via* increased local complement activation and serum carboxypeptidase N-mediated processing of C3a into C3a-desArg (49) (Figure 3). This aligns with the finding that heightened complement activation indeed correlates with the induction of insulin resistance, a cardinal “feature” of severe obesity (50). C1q-deficient mice fed with a high-fat diet accumulated lower levels of complement activation products in their livers and were protected from hepatic insulin resistance (51), while CD55 (decay acceleration factor, DAF)-deficient animals, which lack a key negative complement regulator and have therefore increased complement C3 and C5 activation, were more sensitive to the development of insulin resistance and also showed altered lipid handling and increased adiposity (52). Interestingly though, mice deficient for the only positive complement regulator, properdin, display also increased fat storage upon a high-fat diet and this is associated with decreased energy expenditure and delayed TG clearance but with no changes in glucose uptake (53). Thus, today we acknowledge that complement plays an important role in metabolic organs and tissues such as pancreas, liver, and adipose tissue (54). Furthermore, and similar to the role of intracellular C3a in the homeostatic survival of T cells (see above), controlled, tonic levels of complement activation may contribute to the normal function of these tissues as C3a, C3a-desArg, and C5a *via* all three anaphylatoxin receptors direct adipocyte development (45) and energy regulation (55–57) as well as control of insulin secretion by pancreatic β-beta cells (11, 54) (Figure 3). And also in analogy to what is observed in immune cells, when complement is activated either above or below threshold levels, also cells constituting metabolic tissues can acquire a pathological hypo- or hyperactive phenotype, which may sustain or exacerbate metabolic pathology *via* a detrimental feedback loop.

The contributions of systemic liver-derived versus local complement activation in metabolic disease have rarely been addressed in published studies; however, it is clear that not all effects observed are solely due to systemic complement activity. For example, specifically, adipocytes produce several complement proteins, including FB, factor D (FD, also known as “adipsin”), factor H (FH), C3, C1q, and properdin (53) and dysregulated synthesis of these factors by adipocytes is associated with partial lipodystrophy and loss of subcutaneous adipose tissue of the upper body (58). Therefore, unwanted increased local generation of a range of key complement factors by enlarged adipose tissue (Figure 3) could clearly contribute to a dysbalance in autocrine complement activity (on adipocytes and also subsequently on incoming immune cells) with damaging effects on the environmental metabolic milieu (49).

For an excellent in-depth overview on the role of systemic complement in metabolic disease, please see Ref. (54).

### Intracellular and Autocrine Complement in Metabolism

Similar to the work on the vital role of autocrine-functioning complement in the induction and contraction of human and mouse effector T cell responses, the discovery of the complosome also allowed us to better understand how tightly complement and cell metabolism are interlinked.

Research on the functional cross-connection between immune cell activation, the induction of effector function, and cell metabolism is currently among the “hottest” fields in immunological research—and it is now broadly acknowledged that polarized differentiation of immune cells, triggered by stimulation with distinct activation, stress, and/or danger signals, drives cell-specific remodeling of metabolic pathways. Importantly, distinct metabolic signatures encompassing metabolic enzymes, nutrient sensor and metabolic checkpoint systems, and epigenetic changes in genes coding for proteins involved in metabolic pathways “accompany” distinct phases of immune responses including the non-activated and/or resting state, the adaption response, and the contraction and memory phases (6, 59, 60). Because T cell development and the respective activation states of T cells (naïve, effector, and memory) can be manipulated and monitored relatively easily *in vitro* and *in vivo*, significant progress has been made particularly in understanding how the cell metabolic machinery enables and drives T cell immunity (61). Quiescent T cells generally maintain a status of low glycolytic activity and reduced nutrient intake, using oxidative phosphorylation (OXPHOS) as “tonic survival” energy source, while activated cells increase both glycolysis and OXPHOS as well as nutrient influx and, upon contraction, reduce levels of glycolysis and return to a state of low-level OXPHOS (59). More specifically, in T cells, cognate antigen and costimulation of metabolically quiescent naïve and memory CD4^+^ and CD8^+^ T cells together with specific environmental signals (for example, cytokines) induces proliferation and differentiation into distinct effector populations (helper CD4^+^ T cell subsets Th1, Th2, Th17, Treg cells, and CD8^+^ cytotoxic T cells, respectively) (59, 62, 63). T cell differentiation into effector cells initiates dramatic changes in cellular metabolic pathway usage. A key event during metabolic remodeling in activated T cells is the upregulation of aerobic glycolysis (Warburg effect), which is a prerequisite for growth and expansion and acquisition of effector function (64, 65). Glycolytic metabolites and intermediary pathways that stem from glycolysis are essential for biomolecular synthesis in proliferating cells (64). Activation of T cells also enhances mitochondrial biogenesis, uptake of amino acids (AAs), and glutaminolysis (66, 67). The metabolic checkpoint kinase mechanistic target of rapamycin (mTOR) senses and integrates incoming signals, particularly AA availability, to regulate metabolic adaptation in cells. Activation and lysosomal translocation of mTOR subsequently triggers glycolysis [*via* induction of hypoxia-inducible factor 1α (HIF-1α) that controls the expression of key glycolitic enzymes (68)], OXPHOS, and lipid synthesis toward threshold levels needed for proliferation and differentiation of resting T cells into effector cells (69–71). While the underlying reasons for this are not fully understood, it seems that the production of the key inflammatory cytokine IFN-γ (thus, Th1 induction) poses an exceptionally high glycolytic demand on T cells (65, 72).

Although this notion was not the focus of those studies, earlier work on the role of autocrine anaphylatoxin activity during mouse CD4^+^ and CD8^+^ T cell activation nonetheless suggested already the direct impact of complement on cell metabolism: autocrine engagement of the C3aR and C5aR1 on T cells activate the phosphatidylinositol-4,5-bisphosphate 3-kinase PI(3)K and protein kinase B (PKB, also known as AKT) signaling cascades to promote CD4^+^ and CD8^+^ T cell activation, proliferation, and survival (29, 30). Consequently, circulating T cells from *C5ar1^−/−^* mice have a decreased lifespan (30). However, T cells from these mice were also unable to produce normal amounts of IFN-γ and to generate optimal Th1 responses (30, 73). In retrospect, this “combined” phenotype is unsurprising, given that PI(3)K and AKT are key upstream activators of mTOR (74, 75) and that the PI(3)K–AKT–mTOR axis is fundamental to the activation of glycolysis and anabolic pathways as well as the regulation of cell survival *via* induction of the antiapoptotic B-cell lymphoma 2 protein (BCL2) (76).

The novel role of the complosome in the direct regulation of cellular nutrient influx and sensing (see below) aligns well with the increasing realization that—although initially discovered as pathogen sensors—the ability of the TLRs, the inflammasomes, and complement to recognize an imbalance in normal cell metabolic processes and to help directing reactive responses is of equal importance to the maintenance of cell homeostasis (77, 78). Moreover, normal regulation of cell metabolism by these old systems requires their coordinated functional crosstalk (77). For example, TLR-dependent sensing of the low-level inflammation accompanying a sustained high-fat diet and obesity controls the resistance to insulin (79, 80) and possibly glucose metabolism in neurons (77, 81). Further, metabolites generated during cell proliferation and effector activity (such as high glucose and AA influx and adenosine triphosphate and ROS generation) induce the NLRP3 inflammasome, while metabolites connected with quiescent, contracting, or tolerogenic cell responses inhibit the inflammasome activities (82, 83).

**Figure 4:**
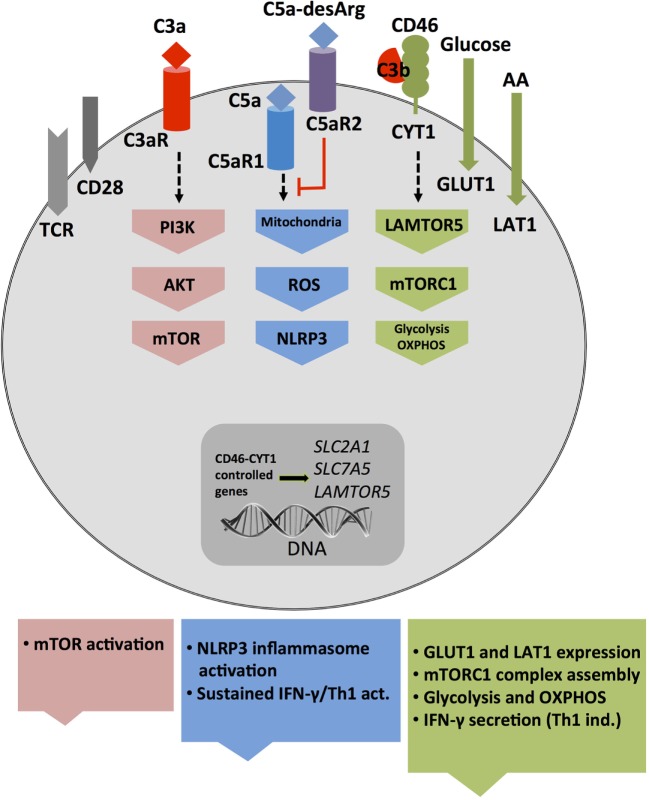
**Autocrine and intracellular complement drives key T cell metabolic pathways during T helper cell type 1 induction**. T cell receptor and CD28-driven autocrine engagement of CD46 CYT-1 (by C3b) leads to upregulation of genes coding for the glucose transporter GLUT (*SLC2A1*), and the amino acid channel LAT1 (*SLC7A5*), allowing for increased influx of glucose and amino acids (AA) into the cell (2). In parallel, CD46 CYT-1-mediated signals induce increased expression of LAMTOR5, and *via* this assembly of the lysosome-based machinery enabling AA sensing *via* mTOR complex 1 (mTORC1), which then leads the induction of glycolysis and OXPHOS required for interferon (IFN)-γ production. Surface activation of the C3aR by C3a activates PI3K and AKT and further sustains mechanistic target of rapamycin (mTOR) activity. Intracellular C5aR1 stimulation (at a not yet defined cellular compartment) drives mitochondrial reactive oxygen species (ROS) production, which induces NLRP3 inflammasome activation and maintenance of IFN-γ secretion during T cell migration into the (inflamed) tissues.

While cell metabolic changes regulate NLRP3 activation and inhibition, it seems that at least in human T cells, complement activation is rather upstream of cell metabolism and a driving force behind sustaining metabolic homeostasis in resting cells as well as mediating nutrient transport during cell activation. For example, the intracellular C3a generated by CTSL-mediated cleavage of C3 within resting T cells drives C3aR signaling on lysosomes and *via* this low level of mTOR activity needed for T cell survival (31) (Figure 2A). Interestingly, this work on intracellular C3 activity led also to the discovery that T (and other) cells from patients with serum C3 deficiency produce normal amounts of intracellular C3a from mutated C3 protein to ensure cell survival but that any C3 activation fragments fail to be secreted (31). TCR stimulation of “healthy” CD4^+^ T cells engages CD46 costimulation *via* surface-shuttled C3b, an event that is critically required for increased expression of the glucose transporter GLUT1 (*SLC2A1*), and the AA transporter LAT1 (*SLC7A5*), that both allow for the heightened influx of “food” in the form of glucose and AAs fueling T cell activation and particularly IFN-γ production (28) (Figure 4). In addition to active regulation of nutrient transport, CD46 also partakes in subsequent intracellular nutrient sensing. CD46-mediated signals were also required for the expression of the late endosomal–lysosomal adaptor, MAPK and MTOR activator 5 (LAMTOR5), a recently described component of the Ragulator complex, which is essential for AA sensing by mTOR complex 1 (mTORC1). In consequence, T cells from CD46-deficient (and serum C3-deficient) patients failed to upregulate GLUT1, LAT1, and LAMTOR5 upon activation and showed diminished mTORC1 activity and IFN-γ production (28, 84) (Figure 4). Conversely, uncontrolled intracellular C3 activation by CSTL and thus hyperactive CD46 signaling occurs in T cells isolated from the inflamed joints of patients with juvenile idiopathic arthritis and underlies exaggerated mTOR activity and IFN-γ secretion in T cells isolated from these patients (31).

Recent work from our group demonstrated that T cells also harbor an intracellular C5 activation system and that signals driven by the intracellularly expressed C5aR1 are required to induce the high(er) levels of oxygen metabolism that sustain particularly Th1 responses after induction (34) as augmented intracellular C5a generation from C5 stores (*via* a yet undefined mechanism) upon TCR and CD46 costimulation induces mitochondrial production of ROS. While CD46 activation simultaneously increases *NLRP3* and *IL1B* gene transcription, the heightened levels of ROS function as a required signal for NLRP3 inflammasome activation and assembly that culminate in the activation of capsase-1 and secretion of bioactive IL-1β (34) (Figures 3 and 4). IL-1β generated by Th1 cells was not required for IFN-γ induction *per se* but needed to sustain optimal levels of IFN-γ during the “dislodge” of T cells from APCs after priming and their migration back into (inflamed) tissues. Thus, *Nlrp3^−/−^* CD4^+^ T cells (in otherwise Nlrp3-sufficient animals) produce only about 50% of IFN-γ upon activation, and this leads to uncontrolled expansion of Th17 cells in the mouse intestine in a T cell transfer model of colitis (34). Interestingly, intracellular and autocrine activity of complement within CD4^+^ T cells is not required for successful Th2 immunity as C3- and CD46-deficient patients mount normal Th2 responses (28, 84). Similarly, only complete absence of CD46-mediated signals reduce IL-17 secretion while a reduction in CD46 expression levels on T cells leads to a proportional reduction in IFN-γ secretion (28). Furthermore, the intracellular C5aR1-induced ROS production drives specifically IFN-γ secretion as suppression of C5aR1 activity or ROS generation fails to affect Th2 cytokine production (34). The reasons for this particularly “intimate” connection between complement and metabolically demanding IFN-γ production are currently unclear but could possibly be rooted in the early co-evolution between complement and metabolism (see Early Coevolution of Complement and Metabolism). The emerging concept that the “complosome” (9), in direct crosstalk with other intracellular effector systems, emerges as critical regulator of normal cell physiology fits indeed well into the scheme that these old immune sensor systems may serve additional and yet to be discovered functions at novel locations: for example, two recent studies describe an unexpected role for the NLRP3 protein in the nucleus—as transcription factor (TF) regulating *Gata3* gene expression in mouse CD4^+^ T cells (85)—and for the secreted NLRP3 inflammasome as extracellular danger signal that amplifies the inflammatory response of macrophages (86).

Importantly, distinct CD46-mediated signals also actively contribute to the metabolic changes that drive Th1 contraction. After successful induction of IFN-γ secretion and Th1 effector function, CD46-mediated signals together with—not yet understood—incoming signals from the IL-2R also induce the switch from a high glycolytic state back to steady-state glycolysis levels in CD4^+^ T cells and, *via* this, subsequently IL-10 co-production and finally Th1 contraction (28, 33). It should be noted that successful CD46-driven IL-10 induction and Th1 contraction also requires a simultaneous decrease in intracellular C5aR1 signaling mediated through autocrine surface C5aR2 engagement as high levels of intrinsic IL-1β production inhibit IL-10 switching (34). This is exemplified by our observation that CD4^+^ T cells from patients with cryopyrin-associated periodic syndrome (CAPS) that have constitutively active NLRP3 due to mutations in the *NLRP3* gene have increased intrinsic IL-1β production and hyperactive Th1 responses that are unable to switch into normal IL-10 co-production (34). While the signaling pathways that mediate the autocrine and intracellular anaphylatoxin receptor functions during Th1 effector responses are ill defined, we know more about the molecular mechanisms by which CD46 regulates cell metabolism. CD46 is expressed in distinct isoforms (due to differential splicing of a single gene) in CD4^+^ T cells and these isoforms differ in the expression of their cytoplasmic domains, termed CYT-1 and CYT-2. Both tails transduce intracellular signals in a range of cell populations (28, 87). Non-activated CD4^+^ T cells express predominantly CD46-CYT-2 (31) while TCR activation leads to the upregulation of the CYT-1-bearing CD46 isoform. And it is CYT-1 that drives the expression of GLUT1, LAT1, and LAMTOR5—all required for mTORC1 assembly and activation with subsequent increased glycolysis, OXPHOS, and IFN-γ secretion (28, 88). The switch from high to low glycolysis and OXPHOS, on the other hand, is mediated by CD46 isoforms expressing CYT-2, which again become the predominant CD46 isoforms in contracting T cells (28, 31). Moreover, the intracellular tails of CD46 are processed by γ-secretase (88), and we demonstrated that nuclear translocation of both domains occurs and that particularly translocation of CYT-1 into the nucleus is required for Th1 induction (28). These observations make it a possibility that CD46 intracellular domains may interact with and modulate the function of TF complexes (possibly as activators and/or inhibitors) and *via* this directly control metabolic pathways. Importantly, CD46 expression in rodents is confined to non-somatic tissues (89) and a complement receptor/regulator or alternative receptor serving as the murine homolog of CD46 in regard to regulation of metabolic programing during Th1 induction and regulation has so far not been identified. Thus, there are substantial differences in the complement-mediated signaling pathways regulating adaptive immunity between species, and this currently poses a hurdle in the straightforward further exploration of CD46’s *in vivo* role in cell metabolism.

In the above sections, we have focused on the direct impact of autocrine and intracellular complement on key metabolic pathways and particularly on T cells as this new functional complosome–metabolism axis has so far best been described in this cell type. However, complement-mediated regulation on metabolism likely occurs in a wide range of cells, clearly engages other (intracellular) effector systems, and impacts on additional cell basic physiological pathways such as cell survival and autophagy. We refer the readers to two recent reviews that cover these important additional subjects in-depth including an outlook into potential novel roles for complement in infection and in diseases with a metabolic “angle” (6, 82).

## Current Substantial Changes in Our Perspectives on the Complement System

The first part of this review summarized published recent developments in the complement field that allow to argue that complement plays unexpectedly vital roles in single cell and organism-wide metabolism. In conjunction with the discoveries of new sites of action (intracellularly) and strong indication that complement also partakes in general basic cell physiology, our perception of this ancient immune surveillance system is now changing. Complement was initially discovered as a serum-active system and was ever since known as the first line of defense against pathogen- and self-derived dangers detected in blood and other body fluids (10, 90). However, the existence of a complosome and its direct ability to regulate cell metabolism suggests that complement may have *functioned originally as an intracellular sensor system and only became a secreted and “systemic” system when life evolved from single cell to multicell and then to multitissue/organ organisms*. Thus, complement may have moved from regulating intracellular physiological (nutrient) balance and cell survival early on towards directing cell autonomous immunity *via* induction of effector function after cell activation and finally into the prime guardian of the extracellular space as we know it today. This view finds support in the recent observations that other classic key immune mediators such as cytokines and the inflammasomes also support very basic cell activities. For example, several cytokines emerged during evolution well before their respective receptors and can directly act as TFs in the nucleus (91). Similarly, the NLRP3 protein also has TF activity and orchestrates the induction of Th2 responses in mouse CD4^+^ T cells *via* directly initiating *Gata3* gene expression (85). And as mentioned above, γ-secretase-mediated cleavage of the intracellular domains of CD46 allows for the nuclear translocation of CYT-1 and CYT-2 where these domains likely directly modulate the activity of TF complexes (28). The early evolutionary appearance of PRRs (see below), their extensive involvement in normal cell physiology, and the strong association with inflammatory and/or metabolic disease states upon dysregulation of these innate immune components (54, 92, 93) lends further argument to the growing understanding that PRRs may not have evolved primarily to protect against infection, but rather as sensors of metabolic changes or imbalances (94). Thus, the second part of this review will now take on a more “perspective-like” focus and explore certain aspects of complement that may support its potential functional origin and emergence from within the cell.

## Early Coevolution of Complement and Metabolism

**Figure 5:**
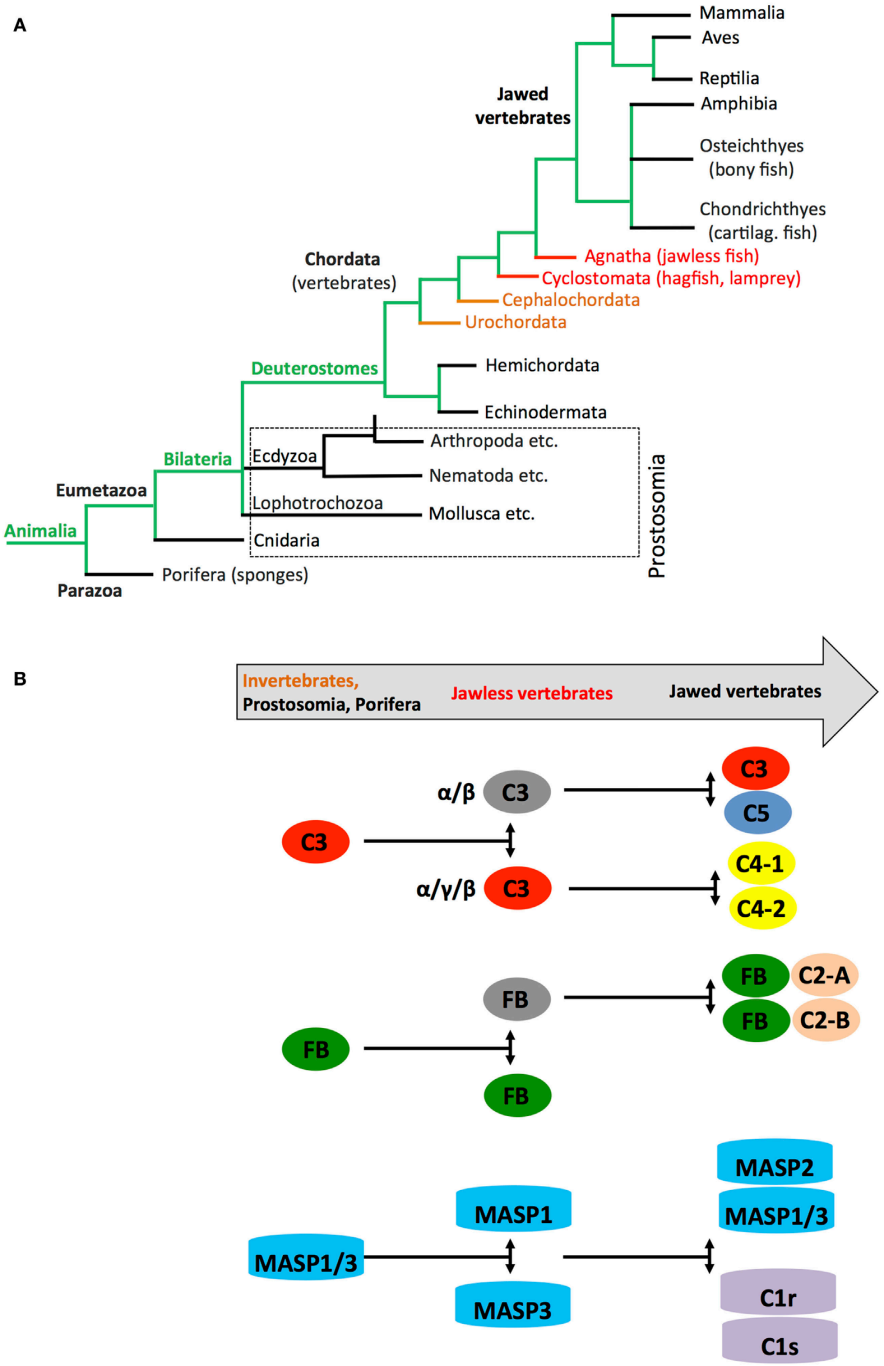
**Schematic depicting appearance of the “original” complement components during evolution**. **(A)** Simplified phylogenic tree of species with emphasis on families and species important during evolution of the complement system (see text). **(B)** Cartoon depicting the appearance of complement(s) C3/C4/C5, factor B (FB), and earliest members of the MASP families during evolution. Color coding in the “title arrow” corresponds to specific key species in the phylogenetic tree under **(A)**. Note that the appearance of fully functional TTC/MAC components and complement receptors and regulators are not depicted in this schematic.

Metabolic activity is the defining characteristic of life. Metabolism evolved from initial oxidation of inorganic matter and CO_2_ in chemoautotrophic organisms about 3.8–2.5 billion years ago to usage of the glycolysis pathways in organisms—probably around 2.4–1.6 billion years ago—and finally to the emergence of OXPHOS around 1.85 billion years ago (95). Glycolysis as means to generate energy is central to almost all known living organisms. However, it was the requisition of mitochondria by cells, and with this, the acquisition of OXPHOS in the presence of an atmosphere increasingly rich in oxygen that was the crucial step in metabolic evolution as this process provided the platform for sufficient energy generation to sustain complex multicellular life (96, 97). Interestingly, the TLRs and the NOD-like receptors appeared when the first primitive multicell layer animals, marine sponges (Demospongiae: Porifera), evolved. Porifera are classified as Metazoa and are considered “precursors” to modern complex animals, which belong to the Eumetazoa clade (Figure 5A) (98). Sponges are filter feeders and have no circulatory systems or organs and are classified as Parazoa (a subkingdom of animals). Similarly, key components of the complement system representing mostly the alternative pathway also appear very early in Cnidaria (Eumetazoa, also see Figure 5A) at the same time as Porifera evolved (32, 99) with C3 (and other thioester-containing proteins (TEPs), see below) and FB already present in sea anemones (100, 101). Subsequent studies on the evolution of the distinct complement components further solidified their early emergence and demonstrated that the complement protein families C3/C4/C5, Bf/C2, C1q/MBL/ficolin, MASP/C1rs, and the TCC/MAC evolved through duplications of core complement genes appearing already in primitive species (102). For example, the genes coding for *C4* and *C5* arose from multiplication of the *C3* gene once in the Urochordates and once in the vertebrates before the emergence of cartilaginous fish (103, 104), while a precursor *Bf/C2* gene appeared before the divergence of Cnidaria and Bilateria (99). The PPR MBL together with MASP1 and 3 are present in the most primitive jawless vertebrates (Agnatha), while this phylogenetic group lacks components of the classical pathway C1q/C1r and C1s (32, 105–107) (Figure 5B). Interestingly, TCC/MAC assembly with fully functional lytic activity seems to also have evolved only later in the higher jawed vertebrate lineage (104, 108). Similarly, effective regulators of complement activation and receptors for complement activation fragments have so far only conclusively been described in bony fish and higher vertebrates (108). However, paired CD18 and CD11 (CR3-like) homologs are present in several Urochordate species and mediate recognition of complement-opsonized antigens (109), while leukocytes of some Agnatha respond to anaphylatoxins—but with the responsible receptors remaining to be identified (110).

Thus, core pathways regulating cell metabolism and complement—or PRRs in general—“met” early in evolution (possibly triggered by the appearance of multicellularity) and it further seems that complement was initially opsonic (and/or metabolic, see below) and only later in evolution became involved in the direct lysis of pathogens.

### The “Metabolic” Domains in the Complement C3 Component

C3 activation is at the very heart of complement function and likely represents the origin of the complement system (111). C3 opsonic activity is mediated by an intrachain thioester bond that, when activated *via* proteolytic cleavage of C3a or activation of C3 *via* hydrolysis (10), attaches C3b/C3H_2_O covalently to surfaces and promotes target opsonization and further complement activation (112). C3 belongs to the TEP family that is divided into two subfamilies: the alpha-2-macroglobulin (A2M) and the C3 subfamilies. In humans, the C3 subfamily comprises C3, C4, and C5, and the A2M family consists of A2M, CD109, pregnancy zone protein (PZP), the PZP-like A2M domain-containing 8 (CPAMD8) protein, and A2M-like protein 1 (A2ML1) (102, 113–115). The A2M subfamily members are characterized by a specific “protease bait domain” and the C3 subfamily members contain an anaphylatoxin (ANA) and *N*-terminal region-like [NTR; also known as C-terminal of C3, C4, and C5 (C345C)] domain. A2M is secreted by the liver, present in high amounts in serum, and functions as an anti-protease as it can trap and inactive a broad range of proteases by covalently attaching to target proteases *via* its thioester (116). While the exact evolutionary origin of TEP proteins remains to be clarified, A2M-like proteins were recently identified in the periplasm of pathogenically invasive bacteria that colonize higher eukaryotes (117).

**Figure 6:**
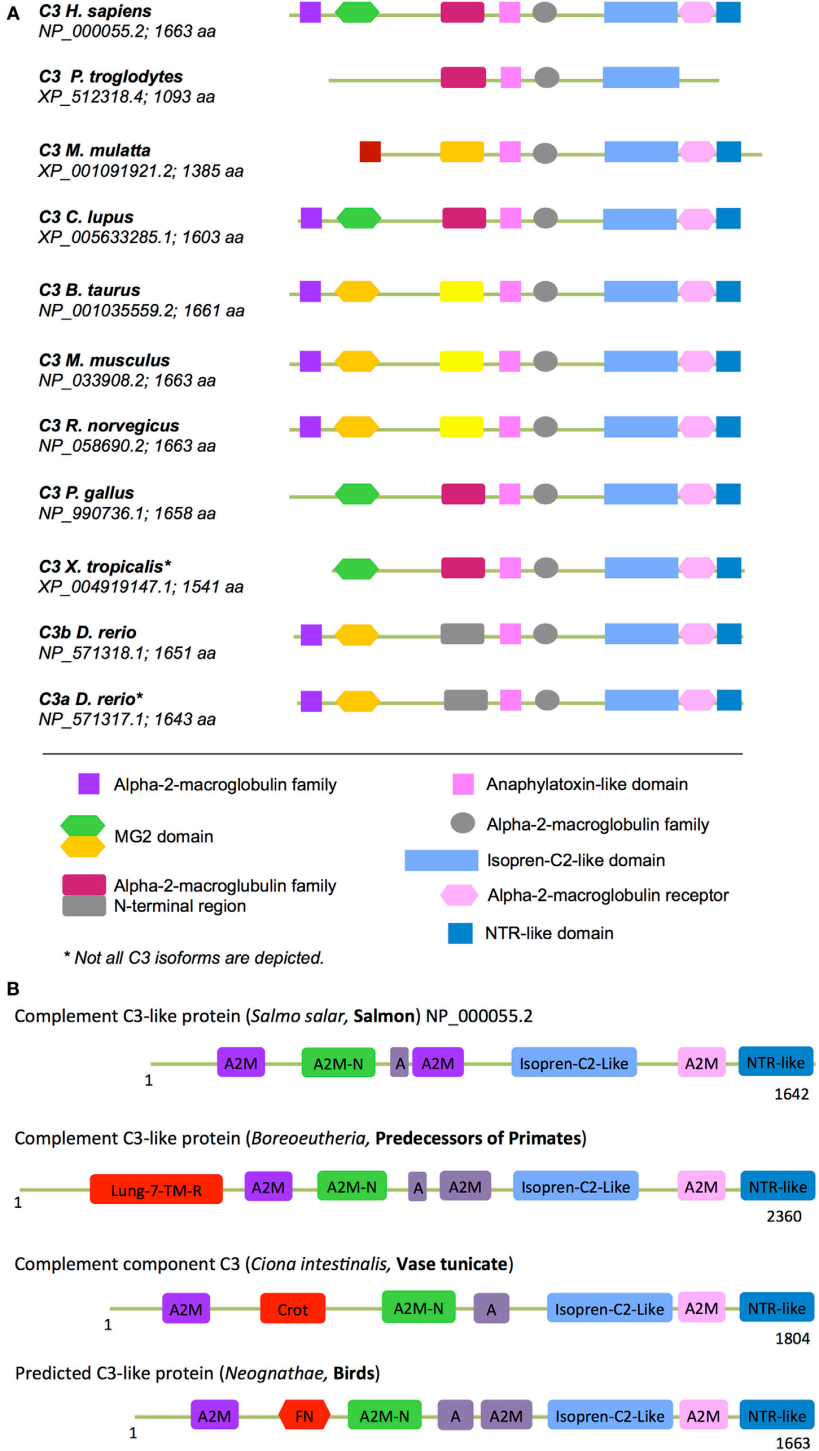
**“Metabolic domains” in modern and ancient C3 forms**. The basic domain structure of C3 is not only conserved among species but also contains several domains that have high homology to proteins with key metabolic functions (see text for further details). **(A)** Compared to C3 found in “modern” higher vertebrates, C3 forms in **(B)** more ancient and basic organisms contained additional domains involved in core metabolic processes. These “lost metabolic” domains are shown in bright red (please see text for details). A, anaphylatoxin-like domain; A2M-N, domain with high homology to the *N*-terminal part of the A2M (alpha-2-macroglobulin) domain; different colors in the A2M domains indicate protein sequence differences among the A2M domains within one C3 form.

In (higher) vertebrates, the C3 structure is highly conserved with C3, C4, and C5 all containing A2M, isopren C2-like domains, and NTR domains (Figure 6A). Interestingly, our analysis of protein domains particularly in the evolutionary older C3 molecules revealed that they often contained additional domains with homology to enzymes and proteins involved in metabolism and/or protein turnover (Figure 6B). “Modern” C3 contains an *isopren C3-like domain* that is commonly found in squalene cyclases and 2,3-oxidosqualene cyclases superfamilies (118). These membrane proteins catalyze a cationic cyclization cascade converting linear triterpenes to fused ring compounds and are thought to be evolutionary precursors to sterol cyclases, which are key enzymes in cholesterol metabolism (119). Similarly, the *NTR-like domain* within C3, C4, and C5 is found in proteins regulating directional axonal growth and cell migration during neural development (120, 121) and in tissue inhibitors of metalloproteinases, which are instrumental in regulating extracellular matrix turnover (122).

Domains found in “older” C3 forms that have been lost in C3 from higher vertebrates (Figure 6B) include the *crotonase* (*Crot) domain*, which is characteristic of members of the crotonase/Enoyl-Coenzyme A (CoA) hydratase superfamily. This superfamily represents a diverse range of enzymes including enoyl-CoA hydratase and dienoyl-CoA isomerase, which catalize the β-oxidation of fatty acids (123, 124). *The 7-lung-transmembrane domain* has homology with GPCRs found in desmosponges, invertebrates, and amoebozoa species (125, 126). The only human protein containing this domain is GPR107. GPR107 localizes to the Golgi and is essential for intracellular vesicular transport (127) and proglucagon mRNA synthesis in pancreatic α-cells (128). The *FNR domain* within C3 from Neognathae can be traced back to proteins found already in ancient anaerobic bacteria and cyanobacteria. This domain is characteristic of ferredoxin NADP(H) reductase (FNR) superfamily members (129) that function as cofactors for flavin adenine dinucleotides, which bind and reduce nicotinamide adenine dinucleotide. FNR was initially identified as catalysator of electron transfer from reduced iron–sulfur protein ferredoxin to NADP^+^, which is the final step in the electron transport (ET) mechanism of the Photosystem I (130, 131). In humans, FNR is found in mitochondria where it serves as first ET protein in the P450 complex and it thus involved in sterol, cholesterol, and steroid metabolism (132, 133).

The presence of these metabolism-related domains in evolutionary older C3/C4/C5 molecules suggests indeed that the complosome–metabolism relationship goes far back and may have contributed to regulation of other key pathways that evolved during the transition from single-cell ancestors to multicellular animals such as those regulating tissue specialization and recognition of self. Thus, it may be worthy to now assess (immune) cell-generated intracellular C3 for novel functions that could possibly include unexpected direct metabolic/enzymatic activities. However, several of these “metabolic” domains in C3 have been lost during the molecular evolution of this family in higher vertebrates while some have been retained. The reasons are unclear, but this could have been driven by the further specialization of C3/C4/C5 into a secreted danger sensing system and the concurrent divergent development of an increasingly complex and “dedicated” metabolic machinery. Although the enzymatic C3 domains could give clues to additional novel functions for complement in metabolism, one should be conscious of these divergence events when studying the role of complement in cell metabolism using different species. Furthermore, several domains within C3 that thought to serve simple structural purpose such as the C1r/C1s, Uegf, Bmp1 (CUB) domain may actually also contribute to novel C3 activities that could involve regulation of metabolic enzymes: the CUB domain in other protein families is involved in oligomerization and recognition of binding partners. Aligning with the idea that the interaction of C3/C3b with other proteins (aside from activation fragment receptors) may go beyond mere target opsonization, we have observed that C3b indeed associates with mTOR and 5′ AMP-activated protein kinase AMPK within cells (unpublished data); however, the functional consequence of this interaction remains to be defined.

### Complement and Mitochondria

While C3 in higher vertebrates may have lost several metabolic domains, it seems that another “younger” complement component that evolved during transition from jawless to jawed vertebrates, C1q, did develop a strong connection to cell metabolism and particularly with mitochondrial function. C1q consists of globular heads that recognize antigen-bound IgM and IgG and a collagen-like stalk region [see Figure 1 and Ref. (134)] and its basic structure is therefore very similar to that of mannose-binding lectin (MBL), one of the initiating molecules of the lectin pathway (105). Apart from its well-defined serum-active functions in complement classical pathway activation and the tagging and clearance induction of apoptotic cells (2), C1q was shown to be present within cells (135, 136) and one of the receptors for C1q, globular head C1q receptor (gC1qR), is located on/in mitochondria (137). Aligning with these data, C1q has been shown to mediate mitochondrial ROS production by cortical neurons in neonatal hypoxic-ischemic brain injury (135). Furthermore, at least 15 hybrid molecules between C1q and the key growth factor tumor necrosis factor (TNF) have been described (138) with most members of this C1q–TNF superfamily displaying metabolic functions. For example, C1q–TNF-related proteins 2, 5, 6, 9, and 13 all increase fatty acid oxidation *via* activation of AMPK (139) and/or acetyl coenzyme A carboxylase (140) in several cell types. In addition, C1q–TNF-related proteins 1, 3, and 9 also activate MAPKs and AKT, which are key upstream initiators of mTOR activity [reviewed extensively in Ref. (141) and see Section “Introduction” above]. C1q–TNF-related protein 3 also drives OXPHOS-supported protein expression and mitochondrial ROS production within smooth muscle cells (142), while exogenous addition of this protein blocked apoptosis of mesenchymal stem cells due to activation of the PI3K–AKT–mTOR axis (143). Importantly, using C1q–TNF-related protein 3 siRNA knockdown technique, the authors of this latter study demonstrated that intracellular and/or autocrine C1q synthesis rather than systemic production was important for its function (142). Finally, and similar to C1q, intracellular C1q–TNF-related protein 3 can bind directly to mitochondrial protein cyclophilin D and protects cells against oxidative stress (144). The existence of these C1q–TNF hybrid proteins is intriguing as TNF itself is a master regulator of a broad range of metabolic pathways in almost all cell subpopulations (145). As most studies on these chimeric molecules have so far focused on the C1q portion, it will be important to also define the TNF domain contributions and to possibly dissect the exact “combined” functions of C1q-TNF hybrid proteins versus those of “standalone” TNF and C1q in mitochondria. Furthermore, patients with homozygous C1q deficiency suffer mostly from SLE and glomerulonephritis, and both diseases have been connected with an increase in deposition of apoptotic cell bodies in tissues due to reduced C1q-mediated clearance of dying cells (146, 147). Given the emerging novel role for intracellular C1q in cell metabolism, an investigation into whether C1q defects could provide additional pathophysiological mechanisms contributing to these disease settings may be warranted.

Mitochondria are thought to originate from ancient α-protobacteria, which invaded archaea-type hosts, and that resulted in their symbiotic relationship, which ultimately led to the emergence of eukaryotic cells approximately 1.5 billion years ago (148). This acceptance of the probacteria by their early hosts could only occur if probacteria would fail to induce a proinflammatory response—and indeed, isolated mitochondria do not evoke significant complement activation (149). “Modern day” bacteria and unicellular eukaryotic organisms, on the other hand, face the opsonic and lytic complement system in serum and have therefore evolved numerous evasion strategies (150–152). For example, *Borrelia burgdorferi* expresses a CD59-analog that blocks formation of the TCC/MAC pore and protects them from lysis (152), and viruses have developed numerous evasion strategies based on the acquisition of complement regulators such as vaccinia complement control protein and monkey pox inhibitor of complement enzymes into their genome [reviewed in Ref. (11, 152)]. Critically, while evolutionary pressure on multicellular eukaryotes resulted in larger and more complex genomes with an increased pool of specialized proteins (153), in bacteria, this pressure led to a streamlining of their respective genomes with loss of non-essential genes and emergence of “multitasking” proteins that served several key functions in parallel (154). This is, for example, exemplified in *Candida albicans*, which has a CR3 analog termed Hgt1p that binds to FH and protects the yeast from complement lysis but that also “doubles” as a high-affinity glucose transporter (155). It is therefore feasible that other complement analog evasion molecules expressed by bacteria, viruses, and unicellular eukaryotes might also have additional simultaneous functions in microbial physiology. Conversely, while the virus-induced and C3-dependent activation of the mitochondrial antiviral signaling protein (MAVS) in human epithelial cells induces protective IFN type I responses during infection (156), we can envision that intracellular C3 activation fragments not only contribute to defense against viruses but possibly also to normal mitochondrial function in resting and activated cells. In this regard, it is noteworthy that the only cell type in the human body lacking mitochondria are erythrocytes (157) and that erythrocytes are also the only cells devoid of CD46 expression (158). It is generally speculated that the “loss” of CD46 expression by red blood cells is a protective measure against fast spread of measles virus [which uses CD46 as cell entry receptor (6)]. However, there could be an alternative explanation pointing to a novel role for CD46 in cell metabolism: intriguingly, avian erythrocytes contain functional mitochondria (159) and their red blood cells do express the CD46 analog molecules CREG and CREM (160). Thus, it would be of interest to assess in the future whether intracellular CD46 (which we have observed in several cells)—possibly *via* engagement through intracellularly generated C3b—may have a yet undiscovered function in mitochondrial biogenesis and/or activity.

Although we are now only slowly beginning to understand and appreciate the close connection between complement, the complosome, and metabolism, its impact could be enormous. This system not only plays a central role in metabolic diseases (6), it is also entirely thinkable now that complement may directly impact on a process that has been a fundamental interest in human philosophy and medicine alike since centuries—aging. Restriction of caloric intake has long been recognized to extend the life span in several animal species, including humans. The underlying mechanisms are not entirely clear, but seem to include a combined effect on reduced cell turnover and autophagy, altered redox balance, and insulin responsiveness (161, 162). Complement is at the nexus of these processes, and several topical studies indeed suggest direct links between complement and aging: C3a downregulates proteasome activity (which is essential to normal protein turnover) in human pigment epithelial cells from older individuals, thereby connecting the complement system with intracellular protein longevity (163); C1q levels increase with age and C1q-mediated activation of canonical wnt signaling—which has been implicated in mammalian aging—promotes age-associated decline in tissue regeneration in mice (164), and C3aR and C5aR activation may regulate telomerase activity and preservation of telomer length in cardiac-resident stem cells (165). Against this background, it will be exciting to now (re)explore the full range of the “powers of this unexpected force from within the cell.”

## Future Perspectives

The unanticipated discovery of the complosome and its key contributions to basic metabolic processes of the cell opened up exciting new avenues to explore novel complement activities, but it also left us with far more questions than answers. From the few insights we have gained so far, it is clear that the location of complement activation (extracellular versus intracellular) dictates its function. However, also the functional outcome of complement activation within cells is likely strongly dependent on the subcellular compartment in which it takes place and where the receptors reside that intracellulary sense and respond to complement activation. Thus, among the many pressing questions that we now raise are, for example: is the composition of the complosome universal or cell specific? Which exact cellular subcompartments contain complement components and receptors and regulators for activation fragments? How is the complosome regulated? How does the complosome intersect with extracellular complement? Finding the answers to these queries will not only enhance our understanding of these rather unexpected facets of this ancient system but also create new opportunities to therapeutically target it.

## Author Contributions

Conceptualization, writing—original draft, and revision: MK and CK.

## Conflict of Interest Statement

The authors declare that the research was conducted in the absence of any commercial or financial relationships that could be construed as a potential conflict of interest.
